# Puerarin prevents high-fat diet-induced obesity by enriching *Akkermansia muciniphila* in the gut microbiota of mice

**DOI:** 10.1371/journal.pone.0218490

**Published:** 2019-06-24

**Authors:** Lei Wang, Yongzheng Wu, Lingjia Zhuang, Xiufang Chen, Haiyan Min, Shiyu Song, Qiao Liang, An-Dong Li, Qian Gao

**Affiliations:** Jiangsu Key Laboratory of Molecular Medicine and Center for Translational Medicine, Medical School of Nanjing University, Nanjing, Jiangsu Province, China; State University of Rio de Janeiro, BRAZIL

## Abstract

Growing evidence indicates that the gut microbiota plays a significant role in the pathophysiological processes of obesity and its related metabolic symptoms in the host. Puerarin, an active ingredient in the root of *Pueraria lobate* has been suggested to have a potent anti-obesity effect. Herein, we tested whether this effect of puerarin is associated with changes in the gut microbiota. In addition to reducing body weight, inflammation, and insulin resistance, puerarin administration significantly altered the composition of the gut microbiota. Notably, puerarin treatment greatly increased the abundance of *Akkermansia muciniphila*, a mucin-degrading bacterium known to be beneficial for host metabolism and significantly downregulated in high-fat diet–fed mice. Further experiments revealed that puerarin increased intestinal expression levels of *Muc2* and *Reg3g* and protected intestinal barrier function (normal permeability) by increasing the expression of ZO-1 and occludin *in vivo* and *in vitro*. These data suggest that puerarin’s enriching effect on *A*. *muciniphila* is mediated, at least in part, by a host cellular response to protect the host from diet-induced metabolic disorders and other diseases.

## Introduction

Obesity as a common precursor of many chronic diseases is associated with low-grade chronic inflammation and intestinal dysbiosis [1, 2]. It can lead to various metabolism-related problems including cardio- and cerebrovascular diseases, type 2 diabetes, chronic kidney disease, musculoskeletal disorders, and digestive diseases [3, 4], therefore represents a major health problem. Studies have revealed that the gut microbiota plays a crucial role in diet-related obesity and glucose and/or lipid metabolism disorders [5, 6]. For example, “obesity-related” and “normal-weight-related” microbiotas exhibit significant differences in their composition and profile and in their ability to utilize glucose and/or lipids in humans and other animals [7, 8]. Moreover, germ-free animals show a significant increase in body weight and metabolic dysfunctions after receiving gut microbiota isolated from donors with high-fat diet (HFD) induced obesity [9], suggesting a causal relation between gut dysbiosis and obesity. The gut microbiota as one of the predisposing factors of metabolic disorders affects the expression of host genes that regulate energy expenditure and storage especially in livers as the intestinal microbiota determines the development of non-alcoholic fatty liver disease in mice [10, 11], whereas, steatosis is among the most common phenotype of HFD mice [12]. More recently, studies revealed that the specific bacterial species *Akkermansia muciniphila* (*A*. *muciniphila*) inversely correlates with obesity and diabetes in both mice and humans [13]. Thus, the gut microbiota may be crucial for metabolic disease etiology.

In fact, the gut microbiota has been suggested as a therapeutic target in various diseases, including obesity and diabetes [14]. Currently, most efforts are focused on fecal microbiota transplantation with encouraging results [15]. Nonetheless, concerns have been raised regarding its safety both at the biological and operative levels [16]. Moreover, the inconsistency in fecal microbiota compositions among donors as well as differences in the procedures are an unavoidable issue [17], in addition to the unregulated state of fecal microbiota transplantation, outside the approved clinical practice [18]. Thus, a simpler and measurable approach, e.g., using food or chemical compounds that are effective and safe for regulating the gut microbiota is desirable.

Puerarin is the major bioactive ingredient in the root of *Pueraria lobate* (Wild) Ohwi and has been known as a combination of medicine and food in traditional Chinese medicine for thousands of years [19]. Puerarin possesses a wide spectrum of biological activities including an anti-obesity effect and has been used in alternative medicine for the treatment of various metabolic disorders [20–22]. Nevertheless, the mechanisms of action of puerarin in these diseases remain elusive.

Herein, we investigated the effect of puerarin on the gut microbiota in mice with HFD-induced obesity. The aim of this study was to establish a link between puerarin’s anti-obesity effect and gut dysbiosis, and to identify the puerarin-induced specific microbiota which may help suppressing obesity. Our results suggest that puerarin is a potent therapeutic agent for gut dysbiosis and provide new evidence for understanding the involvement of the gut microbiota in HFD-induced metabolic disorders.

## Materials and methods

### Ethics statement

Animal experiments were carried out in strict accordance with the recommendations in the Guide for the Care and Use of Laboratory Animals (Nanjing University) and were approved by the Institutional Animal Care and Use Committee of Nanjing University.

### Animals and experimental design

Male C57BL/6 mice (6–7 weeks old, n = 24) were purchased for this study from the Model Animal Research Center of Nanjing University. Prior to our experiments, the animals were provided with feed and distilled water *ad libitum*. They were housed at a facility with a good ventilation and air filtration system at 20 ± 2°C, 50% ± 10% relative humidity, and 12 h light/dark cycle. All procedures were strictly compliant with the rules of the Animal Protection and Use Committee of Nanjing University and consistent with the National Institutes of Health Guide for the Care and Use of Laboratory Animals.

**Fig 1:**
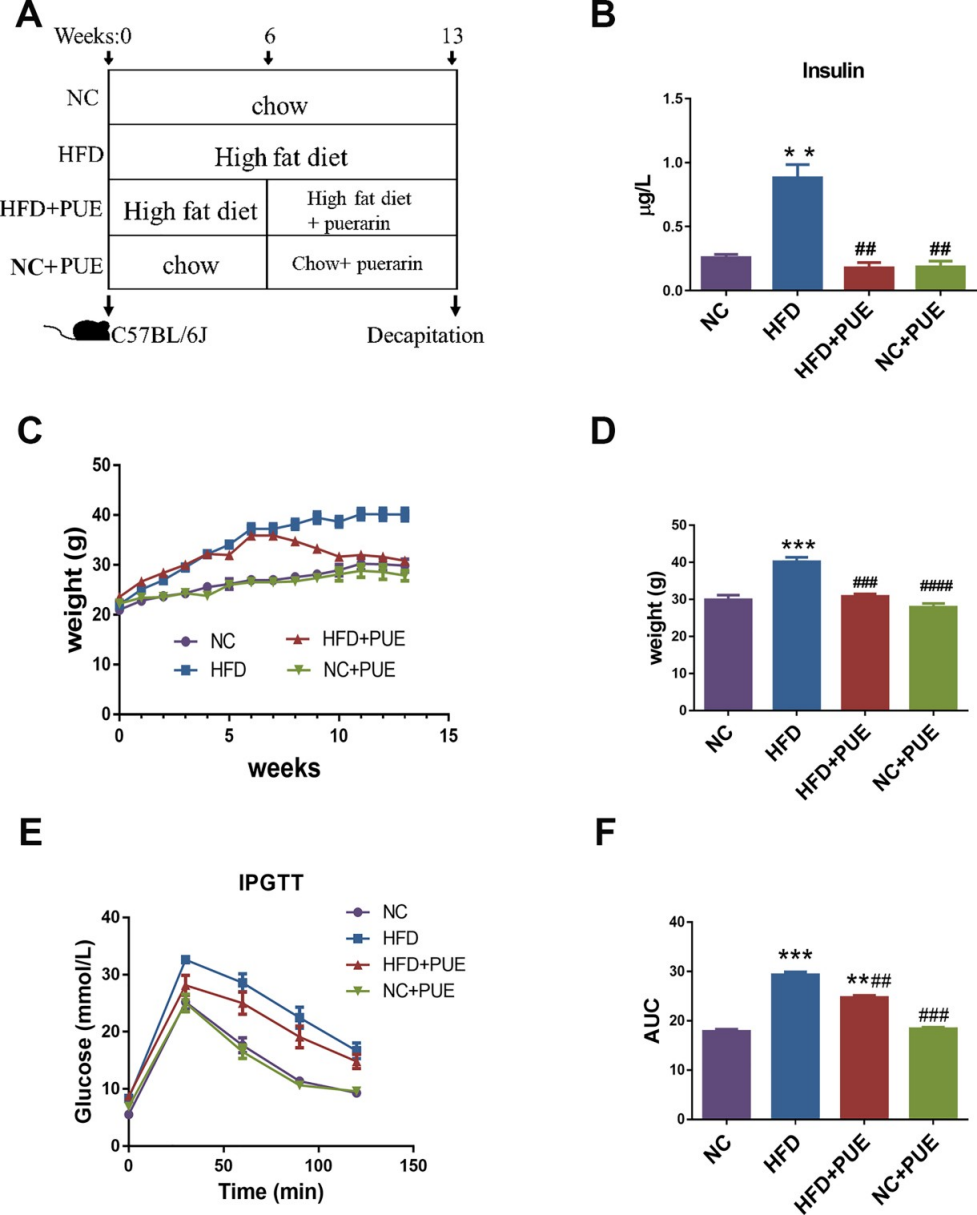
Anti-obesity effect of puerarin in mice fed HFD. (A) A schematic representation of the experiment. (B) Serum insulin concentration (μg/L). (C) IPGTT readouts. (D) The AUC for IPGTT. (E) Body weight (g) of the four groups of mice during 13 weeks. (F) Body weight (g) of the four groups after 13 weeks. Data are presented as mean ± SEM. As compared with the NC group: *P < 0.05, **P < 0.01, and ***P < 0.001. As compared with the HFD group: #P < 0.05, ##P < 0.01, and ###P< 0.001. NC: the control group, HFD: high-fat diet group, HFD+PUE: high-fat diet with puerarin treatment, NC+PUE: control with puerarin treatment.

Mice were randomly divided into four groups (six mice in each group): NC (normal control), NC+PUE (NC+puerarin, 100 mg/kg), HFD and HFD+PUE (HFD + puerarin at 100 mg/kg). A schematic representation of the experimental process is shown in Fig 1A. Mice of the NC group and NC+PUE group were allocated to a low-fat diet containing 10% calories from fat (D12450J; Research Diets, Inc., New Brunswick, NJ, USA) while mice of the HFD group and HFD+PUE group were allocated an HFD containing 60% calories from fat (D12492; Research Diets, Inc., New Brunswick, NJ, USA) for 13 weeks. The composition of the diets was listed in supporting information (S1 and S2 Tables). From the 6th week, mice of the NC+PUE and HFD+PUE groups were daily injected intraperitoneally (i.p.) with puerarin (100 mg/kg), whereas the NC group mice were daily injected with the same volume of vehicle (5% propanediol, i.p.) for 7 weeks. Body weights were recorded weekly. All the animal experiments were repeated twice. Puerarin (batch number 130902) for injection was bought from Baiyunshan Tianxin Pharmaceutical Co., Ltd. (Guangzhou, China).

### Blood glucose measurement and biochemical analyses

For i.p. injection glucose tolerance testing (IPGTT), mice were fasted for 12 h overnight with free access to water followed by an i.p. injection of glucose (2 g/kg body weight; Sigma). Blood glucose levels were measured with a glucometer (Roche) immediately before and 30, 60, 90 and 120 min after glucose injection. Serum insulin concentration and aspartate transaminase (AST) and alanine aminotransferase (ALT) levels were determined by means of commercial ELISA kits (Excell, Shanghai, China) according to the manufacturer’s instructions.

### Gut microbiota analysis

Stool samples were collected from the colon of mice and stored at −80°C before use. DNA was extracted from 0.18 to 0.22 g of each stool sample using the TIANamp Stool DNA Kit (Qiagen, Germany) following the manufacturer’s protocol. The yield and quality of the extracted DNA were measured on a NanoDrop spectrophotometer (Thermo Scientific, USA); one of the PUE group samples (PUE4) with low quality was excluded for the subsequent experiments. The V4 hypervariable region of the 16S rRNA gene was amplified with primers 515F (5′-GTG CCA GCM GCC GCG GTA A-3′) and 806R (5′-GGA CTA CHV GGG TWT CTA AT-3′) [23] for sequencing-library construction. High-throughput sequencing was performed at Novogene Corporation on an Illumina MiSeq platform using the PE250 strategy (paired-end 250 bp sequences).

Determination of relative abundance of *A*. *muciniphila* was carried out according to another publication [24]. Briefly, *A*. *muciniphila*–specific primers (forward: 5′-CAG CAC GTG AAG GTG GGG AC-3′, reverse: 5′-CCT TGC GGT TGG CTT CAG AT-3′) were used to detect *A*. *muciniphila* by real-time PCR.

### Histological analyses and euthanasia concern

The animals were anaesthetized by isoflurane using an anesthetizing chamber (MSS International Limited, West Yorkshire, UK) to a sealed plastic box, and the concentration of isoflurane was monitored continuously using a Vamos gas analyzer (Drager, Lübeck, SchleswigHolstein, Germany) [25]. We used 4% concentration of isoflurane for inducing anesthesia and 2% concentration of isoflurane for maintenance, the flow rate was kept at 0.6–0.8 L/min [26, 27]. The blood was initially collected by the heart punctures in mice. The animals were then sacrificed by cervical dislocation. The liver (the major lobe) and the small intestine (the ileum) tissues were removed from mouse bodies, flushed with PBS and fixed in a 4% paraformaldehyde solution at room temperature. After sectioning, hematoxylin & eosin (H&E) staining and Oil red O staining were performed for histopathological examination. For staining of goblet cells, the tissues of the small intestines (ileum) were recognized and dissected ~1 cm above the cecum (the colon tissue was obtained from the mid-portion of the colon between the cecum and the rectum) and incubated in Bouin’s fixative for 16 h at 4°C and blocked in 2% blocking agar for paraffin embedding. Next, 5~7-μm sections were prepared and stained with Alcian Blue, Periodic acid‐Schiff (PAS), or H&E. The goblet cells stained pink to red, and the nuclei were blue.

### RNA extraction, cDNA synthesis, and quantitative reverse-transcription PCR (RT-qPCR)

Total RNA was extracted from intestinal tissues (ileum and colons, see above) before tissue fixation using the TRIzol Reagent (Bioteke Corporation, China) according to the manufacturer’s protocol, reverse transcribed into complementary DNA (cDNA), and stored at –20°C. For RT-qPCR, cDNA was amplified with the SYBR Green Mix (Vazyme, China) on a Step One Plus real-time PCR system (Applied Biosystems, USA). The relative amount of mRNA or gene toward an internal control was calculated by the 2^−ΔΔCt^ method. The primers used to amplify target transcripts are listed in **Table 1**.

**Table 1:** Primer sequences for quantitative real-time PCR.

Primer		Sequence (5′ to 3′)
PEPCK	Forward	CTGCATAACGGTCTGGACTTC
	Reverse	CAGCAACTGCCCGTACTCC
G6PD	Forward	CACAGTGGACGACATCCGAAA
	Reverse	AGCTACATAGGAATTACGGGCAA
FOXP3	Forward	CCCATCCCCAGGAGTCTTG
	Reverse	ACCATGACTAGGGGCACTGTA
MUC2	Forward	AGGGCTCGGAACTCCAGAAA
	Reverse	CCAGGGAATCGGTAGACATCG
KLF4	Forward	GTGCCCCGACTAACCGTTG
	Reverse	GTCGTTGAACTCCTCGGTCT
REG3G	Forward	ATGCTTCCCCGTATAACCATCA
	Reverse	GGCCATATCTGCATCATACCAG
IL-6	Forward	CCAAGAGGTGAGTGCTTCCC
	Reverse	CTGTTGTTCAGACTCTCTCCCT
IL-10	Forward	GCTCTTACTGACTGGCATGAG
	Reverse	CGCAGCTCTAGGAGCATGTG
MCP-1	Forward	TTAAAAACCTGGATCGGAACCAA
	Reverse	GCATTAGCTTCAGATTTACGGGT
β-actin	Forward	GGCTGTATTCCCCTCCATCG
	Reverse	CCAGTTGGTAACAATGCCATGT

### Western blot analysis

The fresh intestinal tissues (ileum and colons) were washed with PBS, harvested, and lysed with radioimmunoprecipitation assay buffer. Protein concentrations were determined by means of the bicinchoninic acid (BCA) protein assay kit (Thermo Fisher, USA) according to the manufacturer’s instructions. Total protein (50 μg) of each sample was resolved by SDS-PAGE in a 10% gel, followed by western blotting to detect the expression of tight junction proteins ZO-1 and occludin. An anti–ZO-1 rabbit polyclonal antibody (Cell Signaling Technology, USA), anti-occludin rabbit polyclonal antibody (Proteintech, China), and anti–β-actin antibodies (Cell Signaling Technology, USA) were used according to the vendor instructions. ECL Plus Blotting Reagent and a Quality One documentation system were employed to quantify the data (Bio Rad Laboratories, Inc., USA).

### Intestinal epithelial cell culture and treatment

Caco-2 cells were purchased from the Chinese Academy of Sciences (Shanghai, China) and were cultured in the Minimum Essential Medium (Life Technologies, USA) supplemented with 1% of a penicillin/streptomycin solution (Gibco, Grand Island, NY, USA) and 20% of fetal bovine serum (Life Technologies, USA) in a 5% CO_2_ incubator at 37°C. Caco-2 cells were cultured in the medium containing 5 or 10 μM puerarin (Cat. No. P5555, Sigma-Aldrich, USA) or 1.5% of DMSO (vehicle) for 6 h.

### Availability of data and material

The sequence data supporting the results of this article are available at the NCBI under the SRA accession number PRJNA510293.

### Data analysis

Sequencing data on the gut microbiota were analyzed in the Uparse software (Uparse v.7.0.1001) [28]. Sequences with ≥97% similarity were clustered to the same operational taxonomic units (OTUs) and were annotated via the GreenGene Database based on the RDP classifier [29]. Subsequent analysis of α-diversity was performed on the output normalized data, which were evaluated using the Shannon index. Principal coordinate analysis (PCoA) was performed to identify principal coordinates and visualize β-diversity in complex multidimensional data. Functional pathways of the gut microbiota were predicted using PICURSt (Version 1.1.1) [30]. LEfSe (Version 1.0) [31] was employed to identify biomarkers for both species taxonomical analysis and functional pathways via calculation of the linear discriminant analysis (LDA) score among different phenotype groups.

The data are expressed as the mean ± standard error of the mean (SEM). Experimental data were analyzed in GraphPad Prism 5 (GraphPad Software, Inc., La Jolla, CA) by one-way or two-way ANOVA or Pearson correlation. Data with P < 0.05 were considered statistically significant. Each experiment was performed at least three times.

## Results

### Puerarin prevents HFD-induced obesity in mice and provides additional benefits

To assess the effect of puerarin on obesity, chow-fed (NC) or high fat diet-fed (HFD) C57BL/6 mice were treated with puerarin for 7 weeks (NC+PUE and HFD+PUE, respectively) (Fig 1A). The HFD+PUE group of mice experienced a significantly lower body-weight gain than did the HFD group of mice (P < 0.001), whereas puerarin-treated control mice showed no obvious changes in their weights (P > 0.05) (Fig 1C and 1D). Similarly, puerarin significantly ameliorated the hyperglycemia in HFD-fed mice with a significantly higher glucose disposal rate at 30, 60, and 120 min when compared with that in the HFD group (Fig 1E). The area under the curve (AUC) for i.p. injection glucose tolerance testing (IPGTT) was calculated to evaluate the overall glucose exposure (Fig 1F); these data indicated a significant improvement in glucose exposure in puerarin-treated mice as compared with that in the HFD group (P < 0.01). On the other hand, puerarin treatment had no obvious effect on glucose exposure of control mice (P > 0.05). Moreover, puerarin treatment decreased plasma insulin levels in the HFD+PUE group when compared with that of the HFD group (P < 0.001; Fig 1B). Microscopic assessment of liver samples revealed that the steatosis induced by the HFD was notably reduced by puerarin in comparison with that in the pair-fed mice (S1A Fig), and Oil red O staining showed that puerarin treatment decreased liver lipid content in the HFD-fed mice (S1B Fig). We also found that puerarin decreased ALT and AST levels (P < 0.05; S1C and S1D Fig), lowered expression levels of the HFD-induced gluconeogenic genes *G6PD* and *PEPCK* in hepatic tissues (P < 0.05; S1F and S1G Fig), and reduced liver weight in the HFD-fed mice (P < 0.05; S1E Fig). Thus, puerarin treatment provided additional metabolic benefits to the HFD-fed obese mice.

### Gut microbiota profile is sensitive to HFD and puerarin treatment

**Fig 2:**
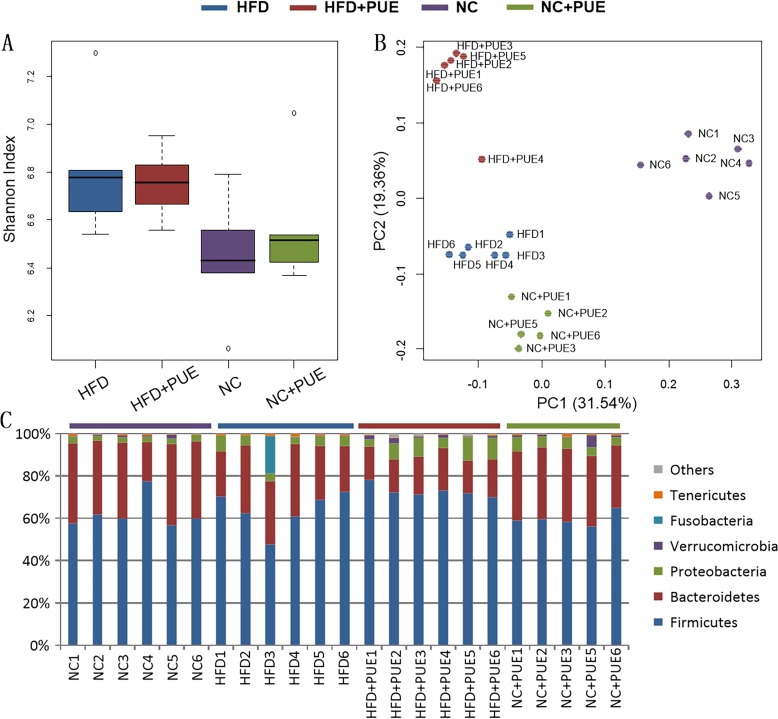
Gut microbiota after puerarin treatment of HFD-fed mice. (A) α-Diversity in all the tested samples; (B) PCoA of the tested samples via the Bray–Curtis algorithm; (C) taxonomic composition at the phylum level.

To test whether the microbiota played a role in the HFD amelioration model in our experiment, the composition and abundance of the intestinal microflora were evaluated by bacterial 16S rRNA gene (V4 region) sequencing in colonic feces samples. A total of 8.8 × 10^4^ ± 2.1 × 10^4^ paired-end reads were generated in the 23 datasets and resulted in 8.5 × 10^4^ ± 2.1 × 10^4^ merged clean reads for the next step of analyses. The Shannon indices of the tested samples are summarized in Fig 2A. The HFD notably increased the diversity of the gut microbiota (P < 0.05), whereas puerarin hardly affected the diversity (P > 0.05). Fig 2B shows the PCoA based on the distribution profiles of all the detected OTUs across the 23 samples using Bray–Curtis ordination, this analysis explained 50.9% of intersample variance for taxonomic profiles. All the 23 samples clustered in accordance with the groups of mice, revealing obvious separation among the gut microbiotas of the four tested groups. These data showed that puerarin’s modulatory effects on the gut microbiota were not limited to the HFD-fed mice.

Fig 2C presents the detected phyla in the tested samples. The HFD significantly increased the abundance of Proteobacteria (from 2.75% ± 0.31% to 4.81% ± 1.32%, P < 0.05) while significantly reducing the abundance of Verrucomicrobia (from 0.75% ± 0.56% to 0.10% ± 0.02%, P < 0.05). Meanwhile, in the HFD-fed mice, the treatment with puerarin notably reduced the abundance of Bacteroidetes and of the phylum Tenericutes (from 27.48% ± 4.94% & 0.69% ± 0.20% to 17.19% ± 1.77% & 0.31% ± 0.03%, respectively, P < 0.05), and significantly increased the relative abundance of the phylum Verrucomicrobia (from 0.10% ± 0.02% to 1.29% ± 0.70%, P < 0.05). In the NC-fed mice, puerarin treatment altered only Proteobacteria abundance (from 2.75% ± 0.31% to 5.03% ± 0.99%, P < 0.05).

### *A*. *muciniphila* inversely correlates with the obese phenotype

**Fig 3:**
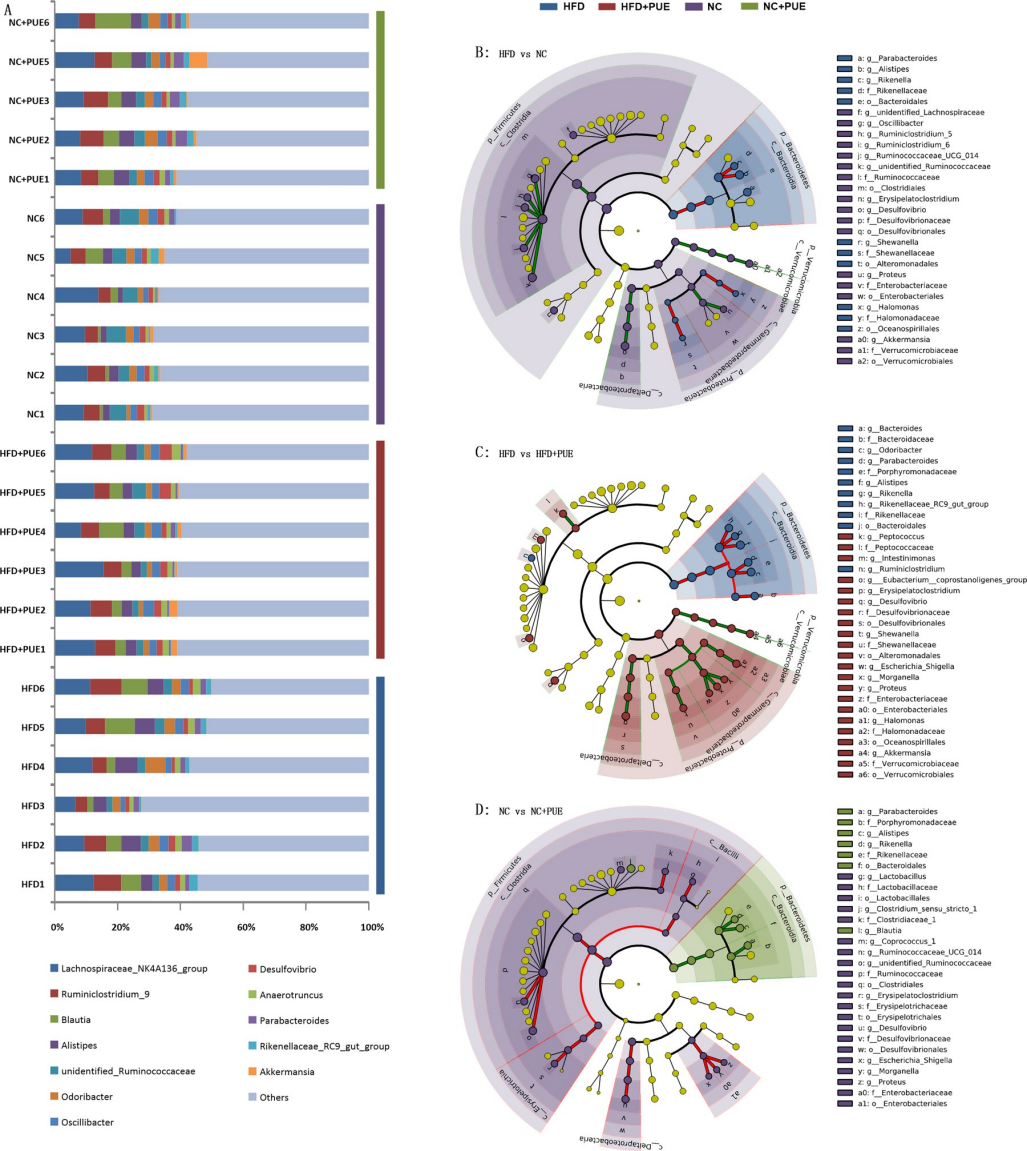
**Taxonomic composition at the genus level (A) and a taxonomic cladogram obtained by the LEfSe analysis of 16S sequences within pairs of groups (B–D)**. The brightness of each dot is proportional to its effect size. Each circle’s diameter is proportional to the taxon’s abundance. HFD vs. HFD+PUE (B), HFD vs. NC (C), and NC vs. NC+PUE (D).

**Fig 4:**
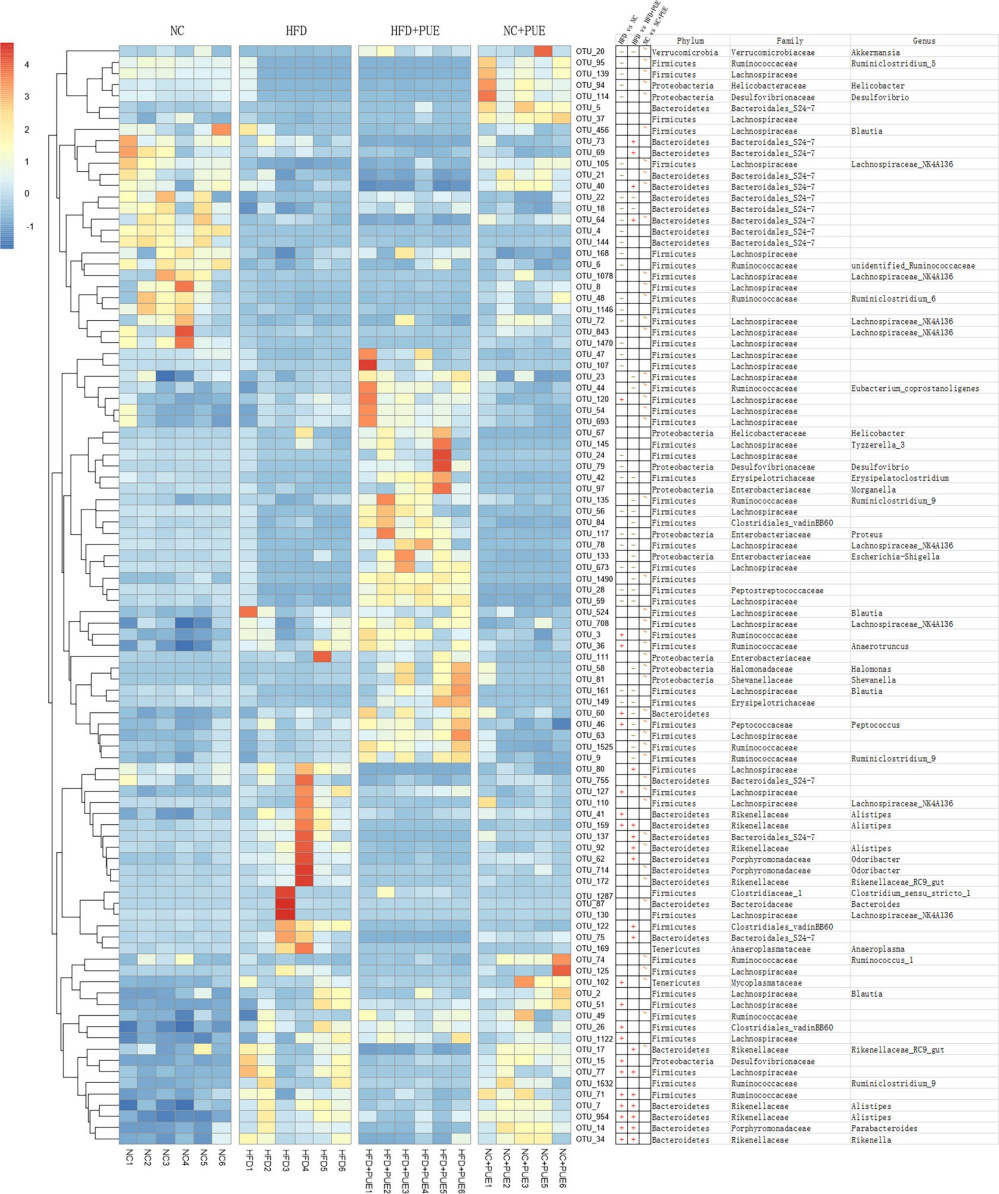
Heatmap of the OTUs with [log_10_] LDA scores >3 in the four murine groups. The *t* test of different pairs of mouse groups: HFD vs. HFD+PUE, HFD vs. NC, and NC vs. NC+PUE (+: significantly enriched in the latter, -: significantly reduced in the latter, ~: no significant difference).

To determine possible correlations of the obese phenotype with the gut microbiota of the tested mice, LEfSe was used to examine the biomarkers of the detected gut microbiota at the genus level by comparing different pairs of mouse groups: HFD versus NC, HFD versus HFD+PUE, and NC versus NC+PUE (Fig 3B–3D). The genera that contributed to differences the most ([log_10_] LDA scores > 2) in the pairwise comparisons are listed in Supporting Information (S2 Fig), where the blue part illustrates the genera that contributed significantly to the difference between the HFD group and NC group or HFD+PUE group ([log_10_] LDA scores > 2). Notably, the genus *Akkermansia* markered obese phenotype with its abundance was significantly lower in the HFD group but was recovered in the HFD+PUE group. To further characterize the relation between the gut microbiota and obese phenotype at the OTU level, increased stringency was imposed ([log_10_] LDA scores > 3) in all the four tested groups, and the remaining OTUs meeting the criteria were summarized (Fig 4); their differences were examined by the *t* test within pairs of groups (HFD vs. HFD+PUE, HFD vs. NC, and NC vs. NC+PUE). Only one OTU that was annotated as *Akkermansia* (OTU_20, Fig 4) manifested significant differences in both comparisons: HFD vs. HFD+PUE and HFD vs. NC (P < 0.05), while showed little difference (P > 0.05) between the NC and NC+PUE groups (S5 Fig). Thus, the abundance of *Akkermansia* and the obese phenotype showed an inverse correlation of changes. Real-time PCR confirmed its identity as *A*. *muciniphila* at the species level (S3 Fig). In short, the abundance levels of *A*. *muciniphila* were significantly enriched (P < 0.05) in the normal-weight mice (NC, NC+PUE, and HFD+PUE).

### Both HFD and puerarin affect functional prediction of the gut microbiota

**Fig 5:**
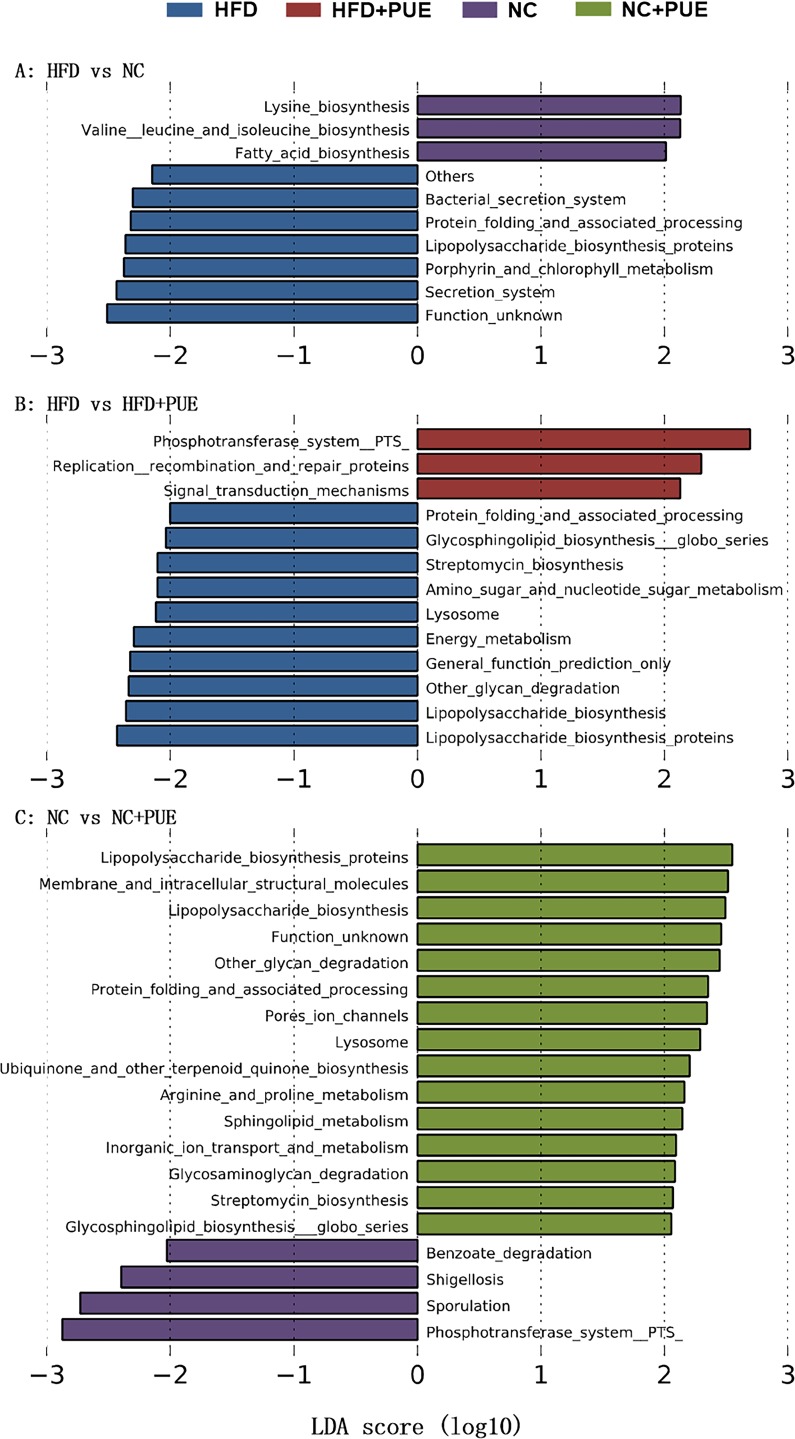
Pathways enriched in pairwise comparisons of groups are indicated with the LDA score. Only taxa meeting an LDA significance threshold of >2 are shown. HFD vs. HFD+PUE (A), HFD vs. NC (B), and NC vs. NC+PUE (C).

The alterations of functional gene abundances in the microbial taxa were predicted based on our 16S rRNA gene datasets by means of PICRUSt (phylogenetic investigation of communities by reconstruction of unobserved state) [25]. As shown in Fig 5, overall, there was no coherent change in the KEGG pathway that was significant ([log10] LDA scores > 2) and consistent with the change of the obese phenotype upon puerarin and/or HFD treatment. Instead, LEfSe did identify two KEGG pathways (lipopolysaccharide biosynthesis proteins and protein folding and associated processing) that were both enriched significantly ([log10] LDA scores > 2) in the groups of HFD-fed obese mice (HFD vs. NC and HFD vs. HFD+PUE) and PUE-treated mice (NC vs. NC+PUE), suggesting that these two pathways might simply characterize the responses of the gut microbiota to the environmental challenges. No KEGG pathway in all the comparisons related to puerarin or HFD treatment showed a significant and persistent reduction.

### Puerarin improves intestinal barrier function (i.e., normalized intestinal permeability) in HFD-fed mice

**Fig 6:**
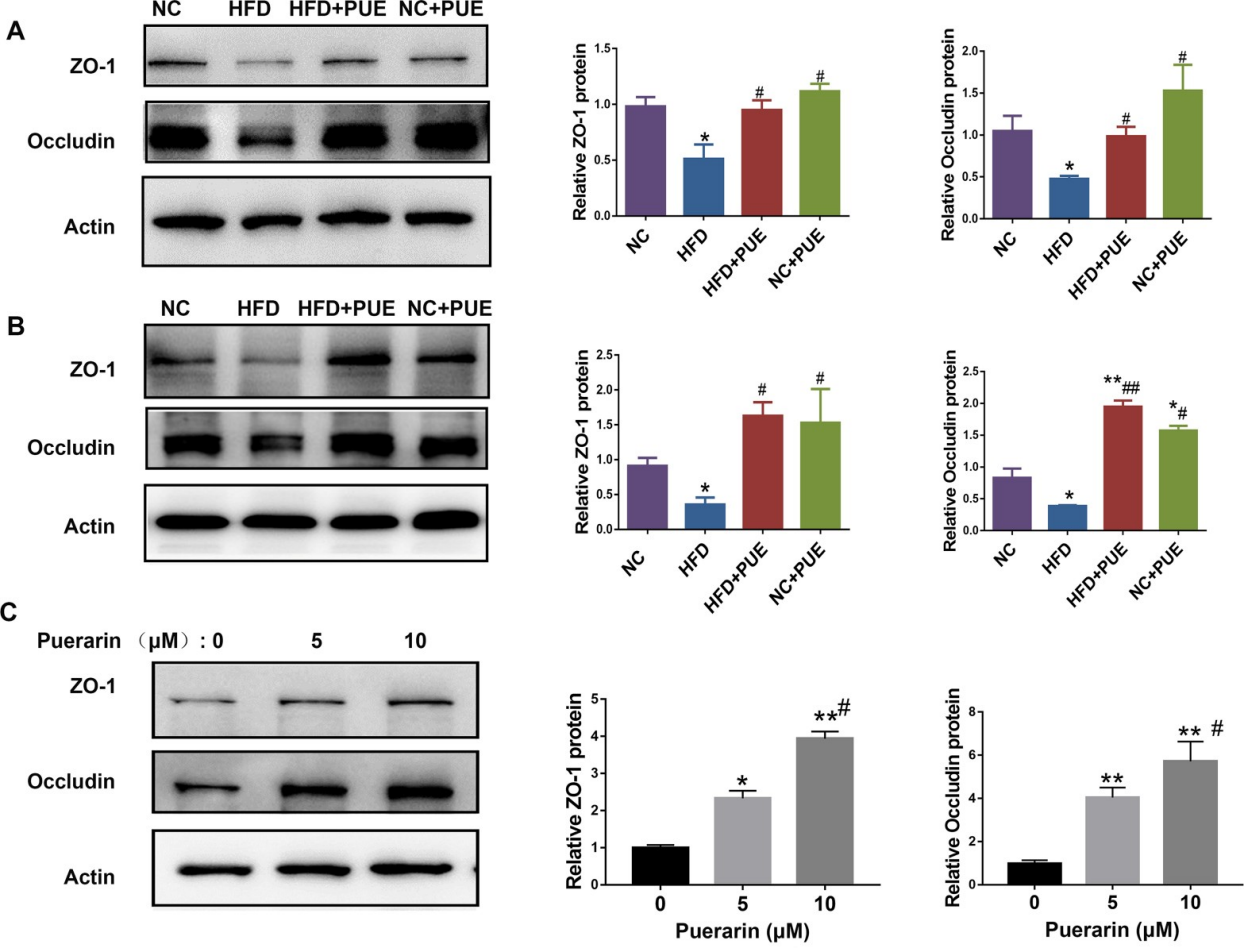
Intestinal barrier was strengthened by puerarin *in vitro* and *in vivo*. (A) Western blot analyses and quantification of the protein expression levels of tight junction proteins (TJPs) ZO-1 and occludin in the small intestine of mice. (B) Western blot analyses and quantification of the protein expression levels of tight junction proteins ZO-1 and occludin in the colon of mice. (C) The protein expression of ZO-1 and occludin in Caco-2 cells with or without puerarin (5 or 10 μM) treatment for 6 h. As compared with the NC group: *P < 0.05, **P < 0.01. As compared with the HFD group: ^#^P < 0.05, ^##^P < 0.01.

Next, to assess the protective effect of puerarin on the intestinal barrier, the expression levels of tight junction proteins ZO-1 and occludin in the small intestine and colon were studied (Fig 6A and 6B). The expressions of ZO-1 and occludin were reduced in the HFD group when compared to those of the NC group, and they were recovered in the HFD+PUE group. To test whether this effect of puerarin was direct, puerarin was incubated with human colonic adenocarcinoma cell line Caco-2 for 6 h (at 5 or 10 μM). The protein levels of ZO-1 and occludin in Caco-2 cells were significantly upregulated compared with those in the cells treated with vehicle (Fig 6C). Moreover, in both the small intestine and colon of HFD-fed mice, puerarin decreased the mRNA levels of proinflammatory cytokines *IL-6* and *MCP-1* and upregulated the mRNA level of anti-inflammatory cytokine *IL-10* (S4A and S4B Fig). In agreement with this result, In HFD+PUE group, Foxp3 expression in the small intestine and colon was significantly increased, which was initially reduced in the small intestine (P < 0.05) and colon (P < 0.01) in the HFD group (S4 Fig). Foxp3 is a critical transcription factor of regulatory T cells, which suppresses immune and inflammatory responses in the intestinal tissues of obese animals [32]).

### Puerarin increases intestinal expression of *Muc2* and *Reg3g*

**Fig 7:**
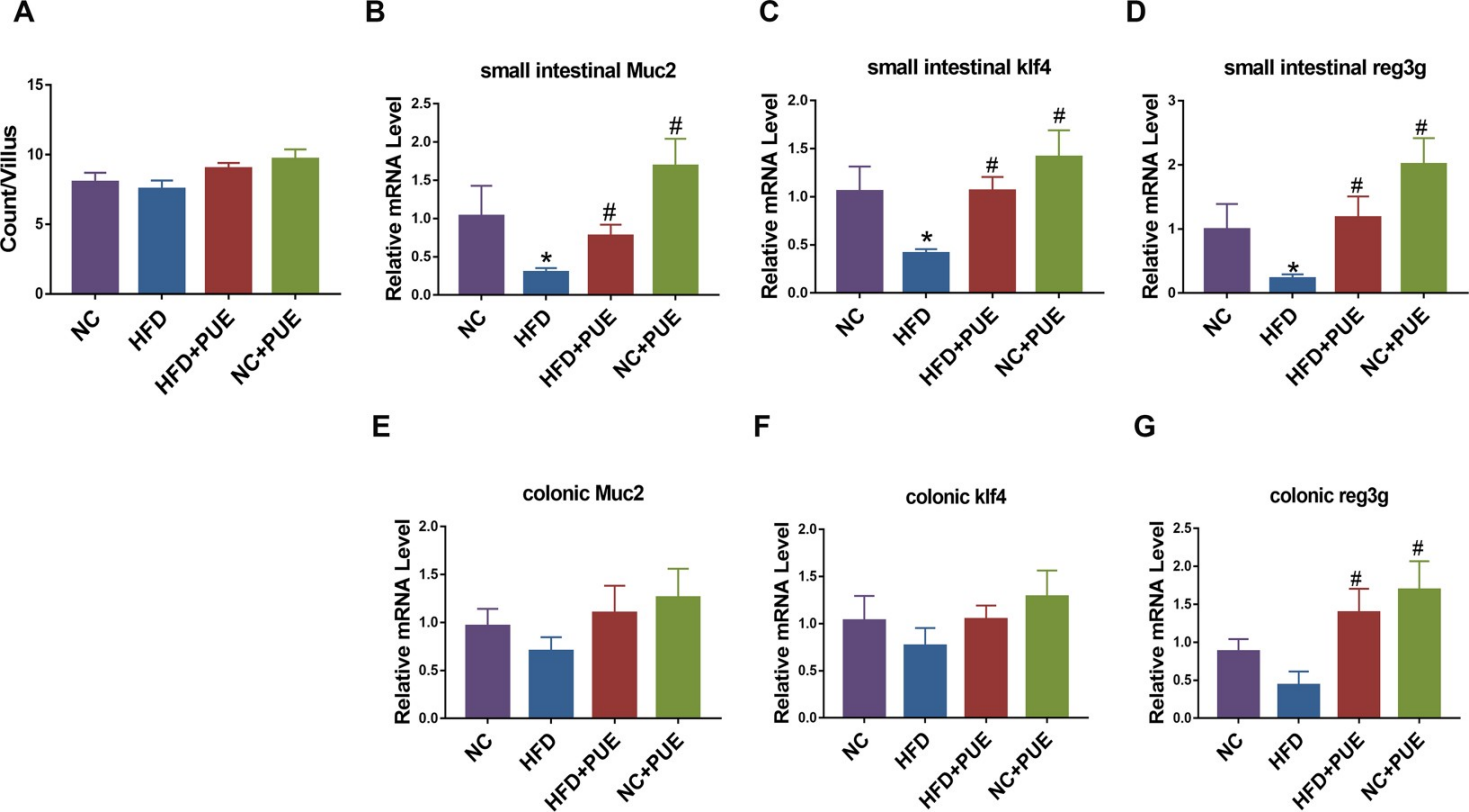
Effects of puerarin on intestinal goblet cells and related genes in mice. (A) Counts of PAS-positive goblet cells per villus. (B–D, E–G) mRNA expression levels of *Muc2*, *Klf4*, and *Reg3g* in the small intestine and colon of mice. Six mice per group. As compared with the NC group: *P < 0.05, **P < 0.01. As compared with the HFD group: ^#^P < 0.05.

Finally, the effect of puerarin on the intestinal goblet cells that synthesize and secret mucins in the intestine (which are known to stimulate *A*. *muciniphila* growth [33]) was evaluated. First, the number of goblet cells were stained with PAS. Although the numbers of PAS-positive cells per villus (8.6 ± 1.6 goblet cells) were not obviously different between the groups (Fig 7A), the expression levels of *Klf4* (a regulator of goblet cell differentiation [34]) and *Muc2* (the major gene encoding mucin in goblet cells [35]) were both significantly lower in the small intestine (P < 0.05, respectively), but not in the colon tissues in the HFD group relative to that in the NC group. The expression levels of *Klf4* and *Muc2* in both NC+PUE and HFD+PUE groups in the small intestines were increased (P < 0.05), indicating a stimulatory effect of puerarin on goblet cell function (Fig 7B, 7C, 7E and 7F). Moreover, the expression of *Reg3g* (which encodes an antimicrobial protein that can restrict bacterial colonization in the mucosal surfaces of the intestines [36]) was also lower in the HFD group and greatly upregulated in NC+PUE and HFD+PUE groups in both the small intestinal and colon tissues (P < 0.05; Fig 7D and 7G). Thus, puerarin promoted intestinal integrity in both the normal and HFD-fed mice.

## Discussion

HFD induced obesity in rodents well mimicked human metabolic syndrome with increase body weight and steatosis, impaired glucose and lipid metabolism, and low- grade inflammation, thus, was broadly used in metabolic disease studies [3–5]. Various models that intended to understand this unwanted chronic condition, mechanistically and pathologically, have been proposed and focused, including glucose/lipid ill-metabolism-driven oxidative stress and fat tissue inflammation [6, 37]. Although, clinic manifestations of metabolic syndrome, e.g. lipid or glucose metabolic disorder dominant, were variable significantly, a low-grade inflammation appears to be a common feature [1, 2]. However, the link between life style and food induced metabolic stress and body inflammation, locally/systemically, remains to be more rigorously established. Recently, gut dysbiosis has been linked to various metabolic disorders, including an obese phenotype, and presumed through impaired intestinal integrity and consequently low-grade inflammation [2], raised the possibility that dysbiosis may be the link between the lipid overloading and the obese condition.

Previously, puerarin has been reported to be effective in preventing an HFD induced obese phenotype by ameliorating impaired glucose and lipid metabolism [38], increasing the energy metabolism in skeletal muscles of obese rodents [39], inhibiting oxidative stress [37], suppressing inflammation [2] as well as improving insulin signaling [40]. However, whether such a broad effect of puerarin on anti-obesity and the related complications is through the regulation of gut microbiota is not known.

Herein, we assess the effect of puerarin on the gut microflora in a mouse model of HFD induced obesity. We showed that puerarin significantly altered the composition of gut microbiota and increased the abundance of Akkermansia muciniphila, a recently recognized key bacteria specie with beneficial effect on diabetes and immune homeostasis [13, 24], along with reversed intestinal barrier dysfunction and inflammation in HFD mice (P < 0.01; Fig 1 and S1 Fig), in addition to its beneficial effects in preventing obesity and the related complications, including steatosis and insulin resistance with no obvious effect on NC mice phenotypically.

It is known that under normal conditions, at the phylum level, Firmicutes (F) and Bacteroidetes (B) constitute the majority (>90% on average) of the gut microbial cells in humans and mice [9]. It has been suggested that the gut microbiotas of obese humans and mice have a notably greater F/B ratio than that of lean counterparts [8, 41]. However, in this study, we (as many others previously [42, 43]) demonstrated that there is no significant difference in the F/B ratio between obese and lean individuals. The contradictory observations indicate that the analysis of the microbiota at the phylum level might not be sufficient for identifying the anti-obesity components of the microbiota [44]. Consequently, exploration of the changes in the gut microbiota at deeper taxonomic levels that are associated with obesity and the related phenotype should be considered [45].

We next showed that, at the family level, the abundance of Enterococcaceae and Bacteroidaceae was increased while the abundance of Verrucomicrobiaceae and Bacteroidales-S24-7 were decreased in the HFD+PUE group of mice as compared with that in the HFD group. Nevertheless, there was still no taxon that could be identified as a biomarker of the obese phenotype in all the four tested groups in this experiment. Moreover, puerarin in normal controls (NC+PUE) also altered the gut microbiota, both at the phylum and family levels, illustrated by the altered Proteobacteria abundance (Fig 2C), with no obvious effect on body weight. Principal coordinate analysis (PCoA) based on the distribution profiles of all detected unique OTUs clustered all the samples in accordance with the experimental manipulations, suggesting that the intestinal flora as whole was sensitive to experimental conditions, but taxonomic profile showed irrelevant to obese phenotype (S5 Fig).

Interestingly, at the genus level, *Akkermansia* was identified as the only biomarker that inversely correlated with the obese phenotype and was enriched by puerarin treatment in HFD+PUE mice, but not obviously changed in NC+PUE mice in the current study, which may explain for the fact that puerarin did not alter the body mass of the control group (NC+PUE mice, S5 Fig). In consistent, in NC+PUE mice, the inflammation cytokines, both pro- and anti-, were not further improved (S4 Fig). So were the Treg marker Foxp3, as well as the epithelial integrity markers (Figs 6 and 7). These results suggested that puerarin had a limited effect on normal control mice. RT-qPCR revealed its identity as *A*. *muciniphila*: the dominant species of *Akkermansia* in the intestine. *A*. *muciniphila* is a gram-negative anaerobic bacterium that degrades mucin and manifests no serious pathogenicity in the intestinal microecological system [46]. It stabilizes host immunity and intestinal homeostasis [13, 47]. *A*. *muciniphila* administration counteracts diet-induced metabolic disorders and mucosal barrier dysfunction and was believed to be beneficial to both type I and type II diabetes conditions at least in animal models [24, 47], and it has been recently used in the treatment of diabetes and obesity [48]. Notably, the suggested beneficial bacterial genus *Faecalibacterium* that was enriched upon oral administration of Gegen Qinlian Decoction, which alleviated type 2 diabetes mellitus [49] was not enriched in our study. This finding suggests that puerarin and Gegen Qinlian Decoction may have different effects on dysbiosis.

Metabolic disorders were characterized by intestinal inflammation and mucosal-barrier dysfunction, which facilitate the translocation of luminal toxicity into the host [50]. An HFD clearly damages intestinal barrier function and downregulates the major goblet cell mucin producing gene *Muc2*, antimicrobial gene *Reg3g* [51], and tight junction proteins ZO-1 and occludin in mice [52]. In contrast, the expression levels of these factors in the intestines were elevated by puerarin administration when compared to that in the HFD group (Fig 6C, 6D and 6F), indicating that puerarin protected the integrity of the intestines.

Furthermore, it has been reported that Tregs are crucial for regulation of obesity-associated inflammation and homeostasis of gut microbiota [53]. The Treg transcription factor *Foxp3* reprograms T-cell metabolism by suppressing glycolysis and by enhancing oxidative phosphorylation and nicotinamide adenine dinucleotide oxidation [54]. In our study, puerarin treatment increased mRNA expression levels of the Treg transcription factor *Foxp3* and anti-inflammatory cytokine *IL-10* but decreased mRNA expression levels of pro-inflammatory cytokines *IL-6* and *MCP-1* (S4 Fig). These data argue that puerarin can regulate Treg generation and host immune balance. These findings suggested that the anti-obesity effect of puerarin may be due to the improvement of intestinal barrier integrity via a reduction in intestinal inflammation and improvement of immunity in HFD mice.

Finally, to counteract low oral bioavailability due to its poor water solubility [55], puerarin was injected i.p. in the present study. Thus, in contrast to gavage, the change in the gut microbiota in the tested mice was more stable and likely, at least in part, due to the direct effect of puerarin on the host cells. Indeed, we observed that puerarin stimulated the expressions of ZO-1 and occludin *in vitro* in the human intestinal epithelial cells, consistent with the observations made *in viv*o. The results confirmed that puerarin can directly act on host cells to deliver the protective effect on gut epithelial integrity. Whether puerarin can target immune cells and functions in metabolic aspect (and regulating the gut microbiota through them) remains to be elucidated.

Mechanistically, in HFD-induced metabolic disorders, the alterations of body mass, glycemia, insulin, and liver condition are all rooted to lipid overloading, which may affect either metabolic process per se or dysbiosis, or both. Here, we presented evidence to argue that puerarin may be through host cells (at least in part) to improve gut epithelial integrity and to reshape gut microbiota. Among the phenotypic changes of HFD mice, the body mass, the liver condition, the insulin level and, perhaps, the T cell homeostasis (indicated by Treg maker Foxp3) were more sensitive to puerarin treatment and largely reversed, while under the current experimental conditions with continuous HFD, the epithelial integrity, the status of the inflammation and the insulin resistance were all only partially rescued, thus, left the possibility that the remaining inflammation, presumably due to an incomplete recovery of gut barrier, is the reason for a compromised rescue of HFD phenotype by puerarin. The complicated mechanistic study is out of the focus of the current work. However, the lessons were learned that to protect diet-related metabolic conditions, both diet restriction and microbiota reshape are necessary and would be more effective.

## Conclusions

We found that the beneficial effect of puerarin on body weight, and insulin level and glucose homeostasis in HFD-fed mice are associated with a change in the gut microbiota with a specific increase in the abundance of *A*. *muciniphila*. This result indicates a potential usefulness of puerarin in clinical practice. Puerarin treatment also promoted the growth and function of goblet cells by increasing the expression of *Muc2* and the antimicrobial-peptide gene *Reg3g*. Thus, we propose that puerarin may prevent the obese phenotype through regulatory effects on the gut microbiota (especially on the abundance of *A*. *muciniphila)* and intestinal integrity through direct effect on the gut epithelial cells.

## Supporting information

S1 Fig Puerarin improves hepatic insulin resistance in HFD-fed mice. (A) Liver H&E staining of four groups of mice (magnification ×200). (B) Liver lipid content was assessed by Oil red O staining (magnification ×200). (C) Quantification of the steatosis percentage in the experimental groups. (D, E) ALT and AST levels in the liver of mice. (F) Liver weight at week 13. (G, H) Relative expression levels of the HFD-induced gluconeogenic genes *G6PD* and *PEPCK* in hepatic tissues were assessed by RT-qPCR. Data are expressed as mean ± SEM; Six mice per group. As compared with the NC group: *P < 0.05, **P < 0.01. As compared with the HFD group: ^#^P < 0.05, ^##^P < 0.01.
(TIF)

S2 Fig LDA scores (log_10_) of the genus manifesting differences within pairs of groups of mice. Only taxa meeting the LDA significance threshold of >2 are shown. NC vs. NC+PUE (A), HFD vs. NC (B), and HFD vs. HFD+PUE (C).
(TIF)

S3 Fig Relative abundance of *A*. *muciniphila* in the feces of mice according to RT-qPCR. Data are shown as means ± SEM (six mice per group). Differences are significant (as compared with the NC group): *P < 0.05. As compared with the HFD group: ^#^P < 0.05, ^##^P < 0.01.
(TIF)

S4 Fig Induction of Foxp3 and reduction in the levels of proinflammatory cytokines attenuate intestinal-tissue inflammation upon puerarin treatment of HFD-fed mice. The mRNA expression levels of *IL-6*, *MCP-1*, *IL-10*, and *Foxp3* in the small intestine (A) and colon (B) of mice. Data represent mean ± SEM. According to one-way ANOVA with the Newman–Keuls *post hoc* test, as compared with the NC group: *P < 0.05, **P < 0.01. As compared with the HFD group: ^#^P < 0.05, ^##^P < 0.01.
(TIF)

S5 Fig Relative abundance of *A*. *muciniphila* in the feces of mice according to the sequencing data on the gut microbiota. Increased stringency was imposed ([log10] LDA scores > 3) in all the four tested groups, and the remaining OTUs meeting the criteria were summarized, the differences were examined by the t test within pairs of groups (HFD vs. HFD+PUE, HFD vs. NC, and NC vs. NC+PUE).
(TIF)

S1 Table The composition of the diets (D12450J; Research Diets, Inc., New Brunswick, NJ, USA) for the NC group and the NC+PUE group. (DOC)

S2 Table The composition of the diets (D12492; Research Diets, Inc., New Brunswick, NJ, USA) for the HFD group and the HFD+PUE group. (DOC)
