# Supplementary figures and images for: Puerarin prevents high-fat diet-induced obesity by enriching Akkermansia muciniphila in the gut microbiota of mice
Source: PLoS One. 2019 Jun 24;14(6):e0218490. doi: 10.1371/journal.pone.0218490 (PMC6590871; doi:10.1371/journal.pone.0218490)

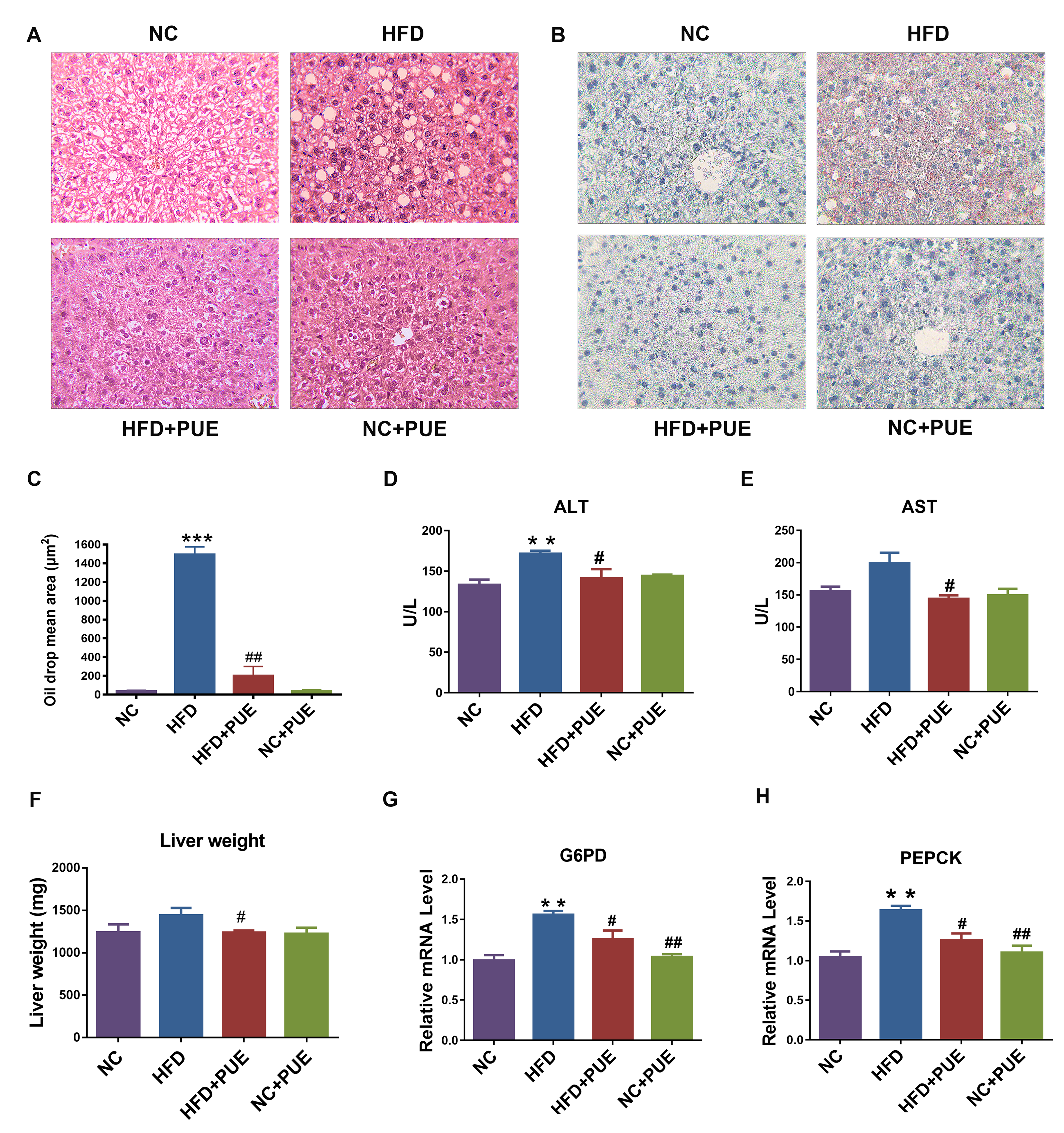

Supplement: S1 Fig — (A) Liver H&E staining of four groups of mice (magnification ×200). (B) Liver lipid content was assessed by Oil red O staining (magnification ×200). (C) Quantification of the steatosis percentage in the experimental groups. (D, E) ALT and AST levels in the liver of mice. (F) Liver weight at week 13. (G, H) Relative expression levels of the HFD-induced gluconeogenic genes G6PD and PEPCK in hepatic tissues were assessed by RT-qPCR. Data are expressed as mean ± SEM; Six mice per group. As compared with the NC group: *P < 0.05, **P < 0.01. As compared with the HFD group: #P < 0.05, ##P < 0.01. (TIF) [file pone.0218490.s001.tif]

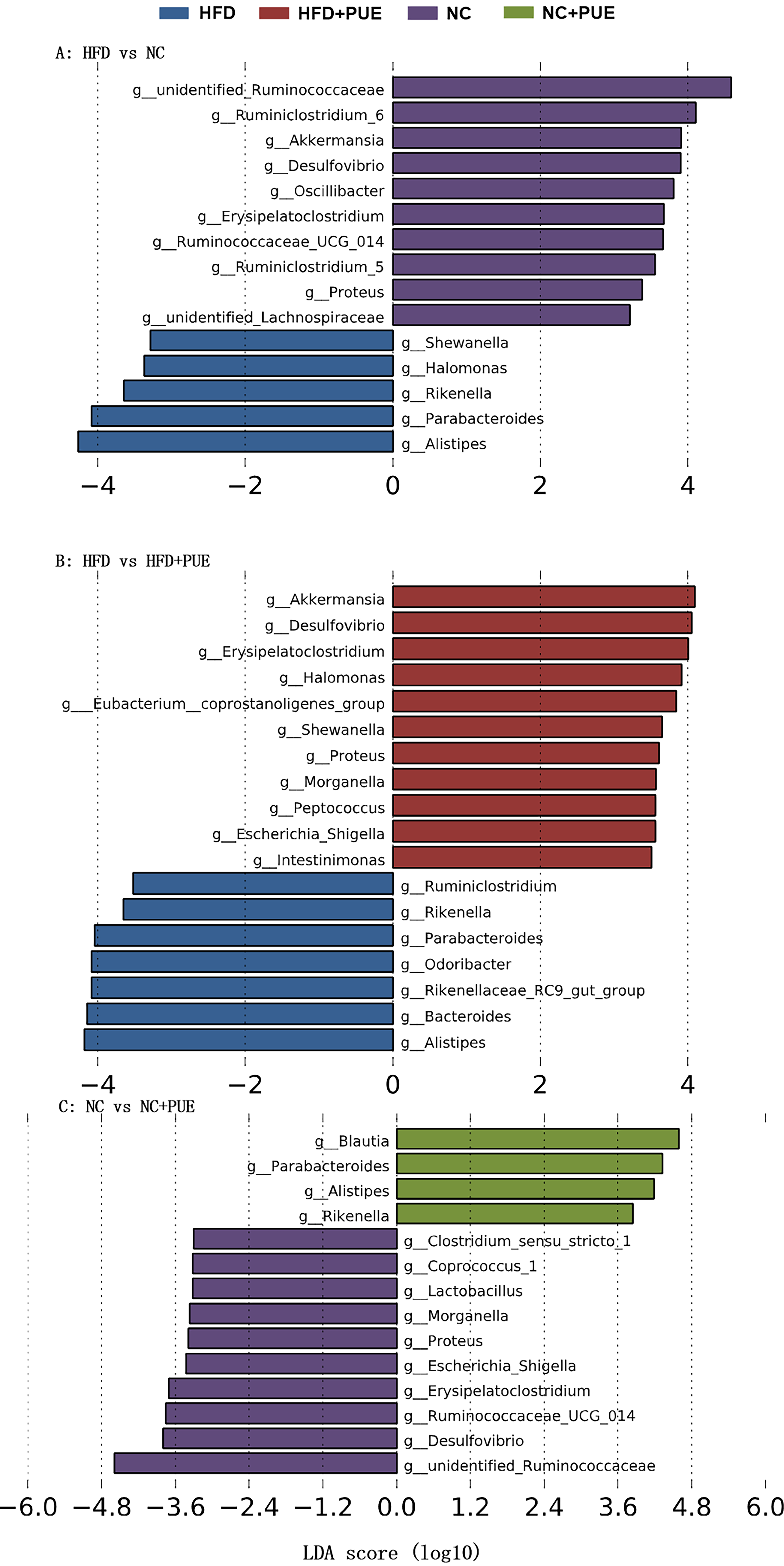

Supplement: S2 Fig — Only taxa meeting the LDA significance threshold of >2 are shown. NC vs. NC+PUE (A), HFD vs. NC (B), and HFD vs. HFD+PUE (C). (TIF) [file pone.0218490.s002.tif]

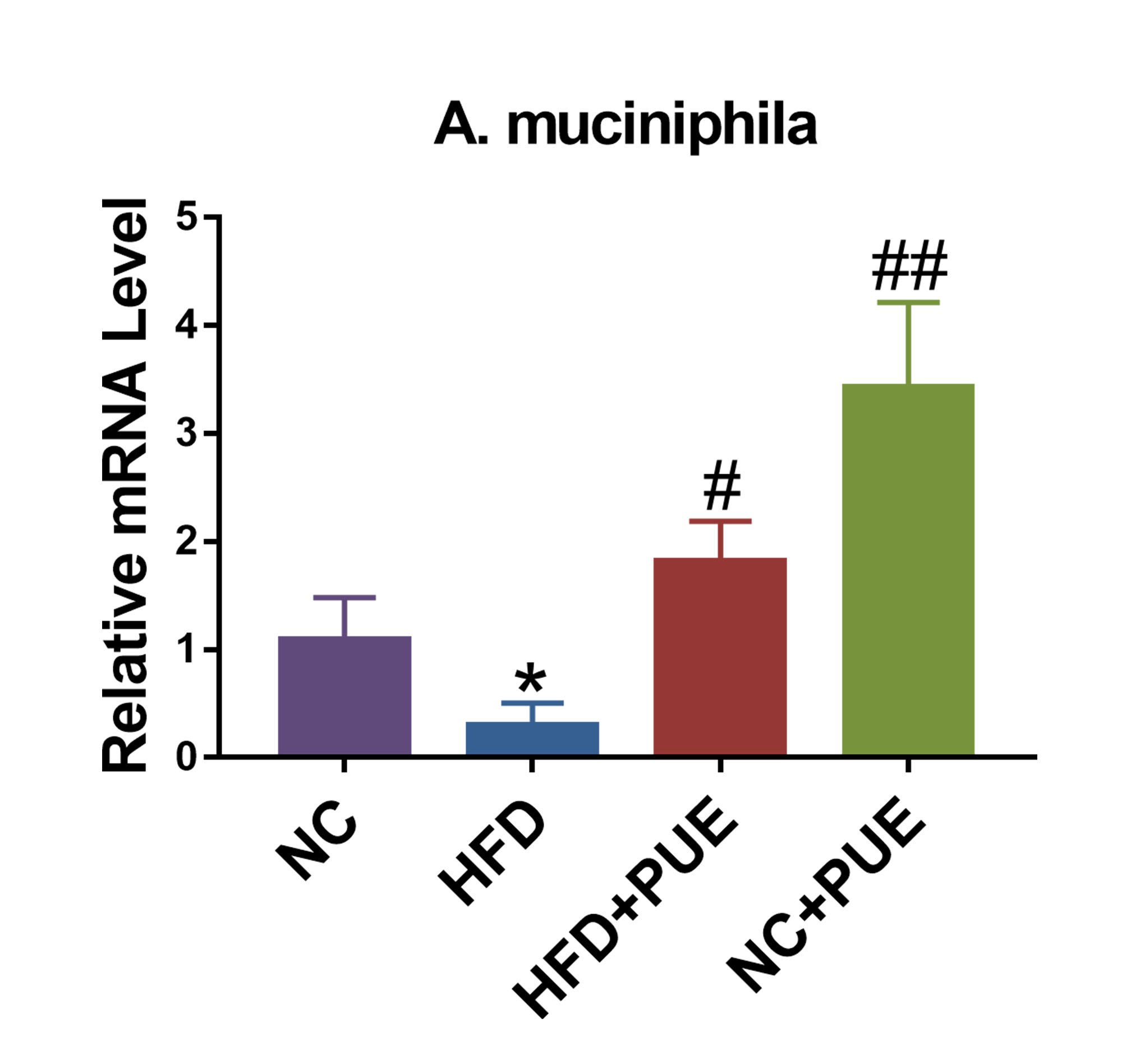

Supplement: S3 Fig — Data are shown as means ± SEM (six mice per group). Differences are significant (as compared with the NC group): *P < 0.05. As compared with the HFD group: #P < 0.05, ##P < 0.01. (TIF) [file pone.0218490.s003.tif]

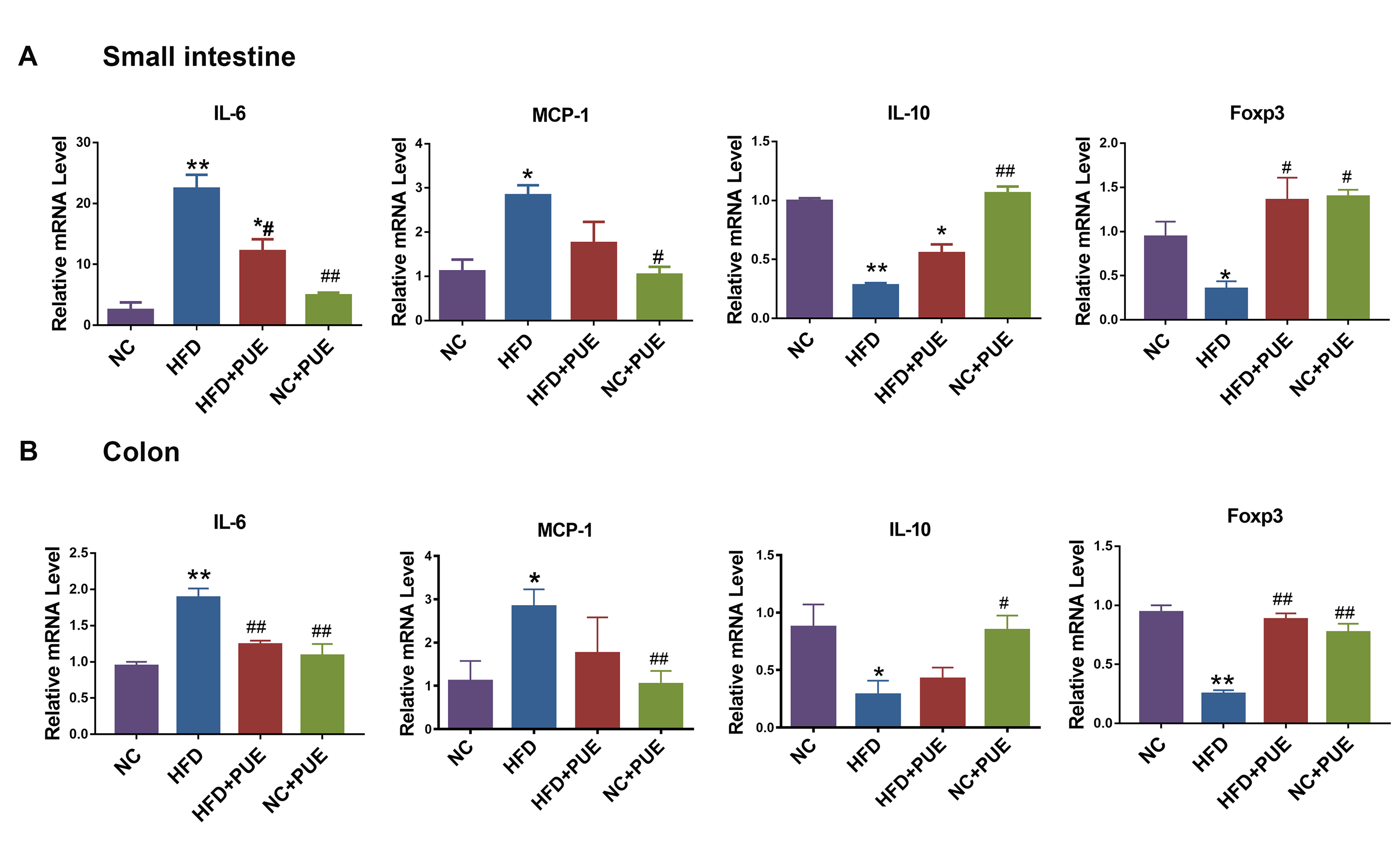

Supplement: S4 Fig — The mRNA expression levels of IL-6, MCP-1, IL-10, and Foxp3 in the small intestine (A) and colon (B) of mice. Data represent mean ± SEM. According to one-way ANOVA with the Newman–Keuls post hoc test, as compared with the NC group: *P < 0.05, **P < 0.01. As compared with the HFD group: #P < 0.05, ##P < 0.01. (TIF) [file pone.0218490.s004.tif]

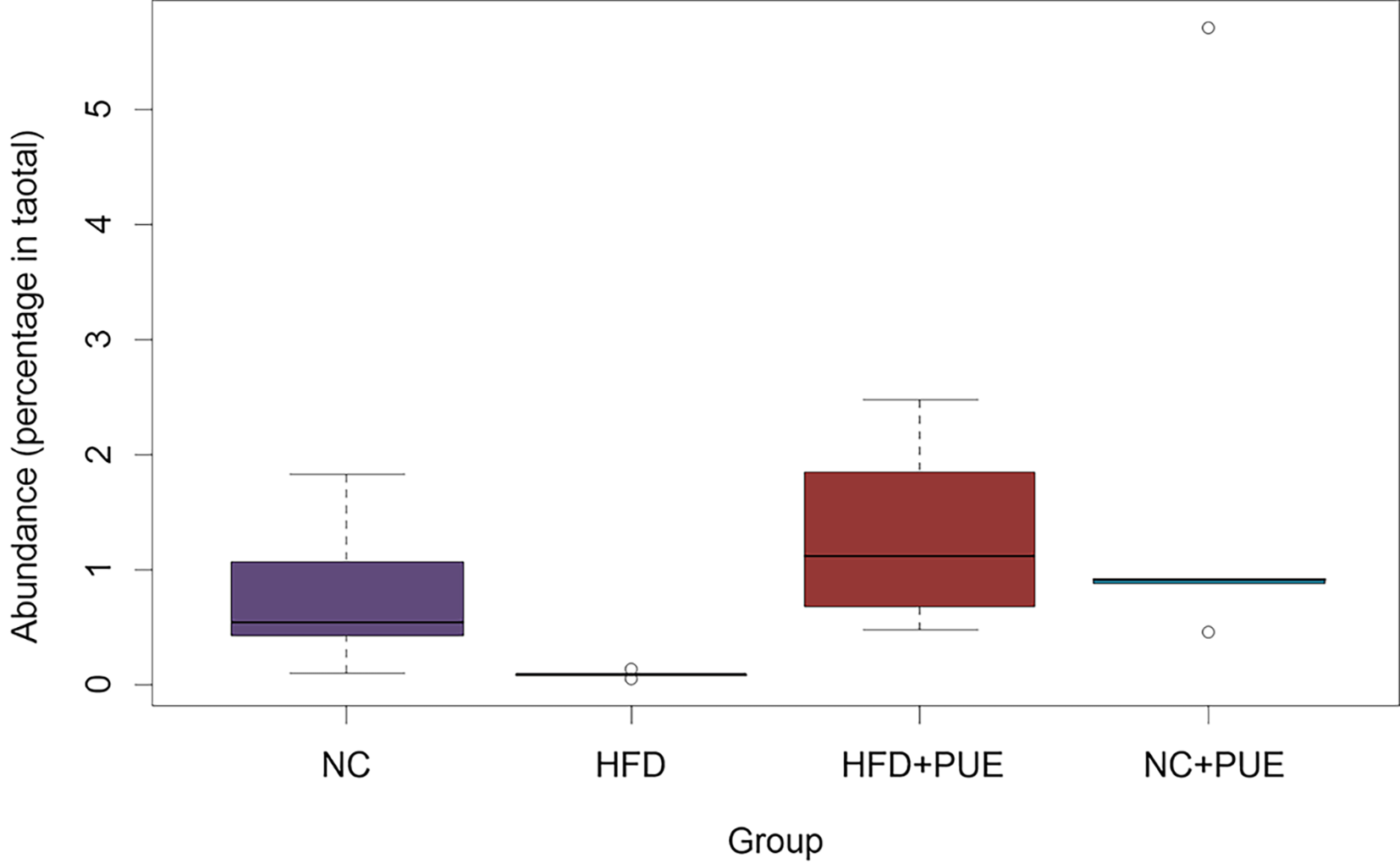

Supplement: S5 Fig — Increased stringency was imposed ([log10] LDA scores > 3) in all the four tested groups, and the remaining OTUs meeting the criteria were summarized, the differences were examined by the t test within pairs of groups (HFD vs. HFD+PUE, HFD vs. NC, and NC vs. NC+PUE). (TIF) [file pone.0218490.s005.tif]
