# Supplementary material for: Puerarin prevents high-fat diet-induced obesity by enriching Akkermansia muciniphila in the gut microbiota of mice
Source: PLoS One. 2019 Jun 24;14(6):e0218490. doi: 10.1371/journal.pone.0218490 (PMC6590871; doi:10.1371/journal.pone.0218490)
Supplement: S1 Table — (DOC) [file pone.0218490.s006.doc]

**Supporting information**

**S1 Table. The composition of the diets (D12450J; Research Diets, Inc., New Brunswick, NJ, USA) for the NC group and NC+PUE group.**

| Class description | Ingredient | Grams | kcal |
| --- | --- | --- | --- |
| Protein | Casein, Lactic, 30 Mesh | 200.00 g | 800 |
| Protein | L-Cystine | 3.00 g | 12 |
| Carbohydrate | Corn Starch | 506.20 g | 2024.8 |
| Carbohydrate | Lodex 10 | 125.00 g | 500 |
| Carbohydrate | Sucrose | 68.80 g | 275.2 |
| Fiber | Solka Floc, FCC200 | 50.00 g | 0 |
| Fat | Soybean Oil, USP | 25.00 g | 225 |
| Fat | Lard oil | 20.00 g | 180 |
| Mineral | Mineral Mix S10026 | 10.00g | 0 |
| Mineral | DiCalcium Phosphate | 13.00 g | 0 |
| Mineral | Calcium Carbonate | 5.5.00 g | 0 |
| Mineral | Potassium Citrate, 1 H2O | 16.50 g | 0 |
| Vitamin | Vitamin Mix V10001 | 10.00 g | 40 |
| Vitamin | Choline Bitartrate | 2.00 g | 0 |
| Dye | FD&C Yellow Dye #5 | 0.04g | 0 |
| Dye | FD&C Blue Dye #1 | 0.01g | 0 |
|  | total | 1055.05g | 4057 |
