# Supplementary material for: Puerarin prevents high-fat diet-induced obesity by enriching Akkermansia muciniphila in the gut microbiota of mice
Source: PLoS One. 2019 Jun 24;14(6):e0218490. doi: 10.1371/journal.pone.0218490 (PMC6590871; doi:10.1371/journal.pone.0218490)
Supplement: S2 Table — (DOC) [file pone.0218490.s007.doc]

**Supporting information**

**S2 Table. The composition of the diets (D12492; Research Diets, Inc., New Brunswick, NJ, USA) for the HFD group and HFD+PUE group.**

| Class description | Ingredient | Grams | kcal |
| --- | --- | --- | --- |
| Protein | Casein, 80 Mesh | 200.00g | 800 |
| Protein | L-Cystine | 3.00g | 12 |
| Carbohydrate | Maltodextrin 10 | 125.00g | 500 |
| Carbohydrate | Sucrose | 68.80g | 275.2 |
| Fiber | Cellulose, BW200 | 50.00g | 0 |
| Fat | Soybean Oil | 25.00g | 225 |
| Fat | Lard Oil | 245.00g | 2205 |
| Mineral | Mineral Mix, S10026 | 10.00g | 0 |
| Mineral | DiCalcium Phosphate | 13.00g | 0 |
| Mineral | Calcium Carbonate | 5.50g | 0 |
| Mineral | Potassium Citrate, 1 H2O | 16.50g | 0 |
| Vitamin | Vitamin Mix, V10001 | 10.00g | 40 |
| Vitamin | Choline Bitartrate | 2.00g | 0 |
| Dye | FD&C Blue Dye #1 | 0.05g | 0 |
|  | Total: | 773.85g | 4057 |
